# Educational level, attention problems, and externalizing behaviour in adolescence and early adulthood: the role of social causation and health-related selection—the TRAILS study

**DOI:** 10.1007/s00787-021-01913-4

**Published:** 2021-11-19

**Authors:** Heiko Schmengler, Margot Peeters, Gonneke W. J. M. Stevens, Anton E. Kunst, Catharina A. Hartman, Albertine J. Oldehinkel, Wilma A. M. Vollebergh

**Affiliations:** 1 Department of Interdisciplinary Social Science, Utrecht Centre for Child and Adolescent Studies, Utrecht University, Padualaan 14, 3584 CH Utrecht, The Netherlands; 2 Department of Public and Occupational Health, Center for Health Inequality Studies, Amsterdam UMC, University of Amsterdam, Amsterdam, The Netherlands; 3 Department of Psychiatry, Interdisciplinary Center Psychopathology and Emotion Regulation, University Medical Center of Groningen, University of Groningen, Groningen, The Netherlands

**Keywords:** Externalizing behaviour, Attention problems, Education, Social causation, Health-related selection

## Abstract

Social causation and health-related selection may contribute to educational differences in adolescents’ attention problems and externalizing behaviour. The social causation hypothesis posits that the social environment influences adolescents’ mental health. Conversely, the health-related selection hypothesis proposes that poor mental health predicts lower educational attainment. From past studies it is unclear which of these mechanisms predominates, as attention problems and externalizing behaviour have the potential to interfere with educational attainment, but may also be affected by differences in the educational context. Furthermore, educational gradients in mental health may reflect the impact of ‘third variables’ already present in childhood, such as parental socioeconomic status (SES), and IQ. We investigated both hypotheses in relation to educational differences in externalizing behaviour and attention problems throughout adolescence and young adulthood. We used data from a Dutch cohort (TRAILS Study; *n* = 2229), including five measurements of educational level, externalizing behaviour, and attention problems from around age 14–26 years. First, we evaluated the directionality in longitudinal associations between education, externalizing behaviour, and attention problems with and without adjusting for individual differences using fixed effects. Second, we assessed the role of IQ and parental SES in relation to attention problems, externalizing behaviour, and educational level. Attention problems predicted decreases in education throughout all of adolescence and young adulthood. Differences in parental SES contributed to increases in externalizing behaviour amongst the lower educational tracks in mid-adolescence. Childhood IQ and parental SES strongly predicted education around age 14. Parental SES, but not IQ, also predicted early adolescent attention problems and externalizing behaviour. Our results provide support for the health-related selection hypothesis in relation to attention problems and educational attainment. Further, our results highlight the role of social causation from parental SES in determining adolescent educational level, attention problems, and externalizing behaviour.

**Supplementary Information:**

The online version contains supplementary material available at 10.1007/s00787-021-01913-4.

## Background

Externalizing behaviour and attention problems have been associated with socioeconomic adversity over the life course [1, 2]. These associations begin in childhood, when children growing up in lower SES households are more likely to show these problem behaviours and subsequently to be assigned to lower educational tracks in selective educational systems [3–5]. Selective educational systems are defined by an early selection into different educational tracks and thus different social contexts, while allowing for mobility between tracks post-selection [6, 7]. Externalizing behaviour comprises a wide range of disruptive behaviours, including overt aggressiveness, as well as more covert rule-breaking (delinquent) behaviours [8, 9]. Externalizing behaviour is often comorbid with attention problems, and both syndromes share common risk factors (e.g. low effortful control, low parental SES) [3, 4, 10, 11]. Both attention problems and externalizing behaviour can be highly disruptive in educational contexts, hence predisposing to academic problems [12–14] and lower educational attainment and SES in young adulthood [1, 15–17]. At the same time, the social environment at school can have a considerable impact on both problem behaviours [18]. Despite these shared features, attention problems and externalizing behaviour involve different behaviours that may affect and be affected by the environment in different ways. Therefore, attention problems and externalizing behaviour might be linked to educational outcomes through different mechanisms, and hence need to be studied separately [8].

Two mechanisms may contribute to the entrenchment of educational inequalities in attention problems and externalizing behaviour: social causation and health-related selection [19, 20]. The health-related selection hypothesis posits that health problems, including problem behaviours, can lead to a decline in educational level [21]. Adolescents with high levels of attention problems experience difficulties following teachers’ instructions and staying on-task in class, as well as when completing their homework. As a result, these adolescents achieve lower grades, have higher risks of repeating classes, and may eventually decline in their educational level [22, 23]. Also externalizing behaviour may interfere with adolescents’ education, as adolescents with high levels of externalizing behaviour often experience rejection by peers with low levels of externalizing behaviour and may gravitate towards academically unengaged aggressive or delinquent classmates. This may lead to a loss of interest in school and a further escalation in externalizing behaviour, which, in its more extreme forms, can lead to expulsion from class, and eventually to a decline in educational track [24].

As opposed to health-related selection, social causation explanations emphasize the role of differences in the social environment in explaining educational differences in externalizing behaviour and attention problems. The selective educational system in the Netherlands entails that Dutch students grow up in distinct educational environments that are characterized by different social norms, future expectations, cognitive resources, and occupational prospects [6, 7, 18]. The lower educational tracks focus on skills training, and often lead to lower-paid vocations that carry less societal esteem than vocations that require attending a higher, more theoretically focused, educational track [6, 18]. Students in the lower tracks may therefore be less able to seek a socio-occupational position valued by society in terms of secure, well-paid, and esteemed employment [18]. These adolescents may then turn to alternative social fields to gain recognition, which may involve affiliation with delinquent peers and externalizing behaviour, as rebellion against meritocratic social norms [25]. Furthermore, peer contagion effects may contribute to stronger increases in externalizing behaviour in the lower educational tracks. Adolescents with long-term behavioural problems are overrepresented in these tracks [15], possibly resulting from health-related selection effects, as discussed above. As a result of this, normatively developing adolescents in the lower tracks are more frequently exposed to externalizing behaviour in the classroom context. Studies have shown that adolescents with initially low levels of externalizing behaviours often mimic the behaviours of aggressive/antisocial peers, themselves becoming more aggressive over time [26, 27]. These peer contagion effects may tilt classroom norms in the lower tracks more strongly toward externalizing behaviours [26, 27]. A higher prevalence of externalizing behaviours amongst the lower tracks may also lead to noisier and unrulier classrooms [28, 29], potentially contributing to higher levels of attention problems in those tracks.

Furthermore, it is pivotal to evaluate the social causation and health-related selection hypotheses in relation to stable background characteristics, which are already present in childhood and predict later educational level, attention problems, and externalizing behaviour. These risk factors may act as time-invariant ‘third variables’ (i.e. confounders) in longitudinal associations between educational level and problem behaviours, and therefore need to be adjusted for in statistical models. From a social causation perspective, family SES is especially important. Lower SES parents may have fewer resources to support the academic performance of their child, leading to a lower initial placement in the educational system [5]. Additionally, these parents often lack the financial means to provide a safe and stimulating environment, which is highly important for children’s cognitive and emotional development [30, 31], leading to increased risks of attention problems and externalizing behaviour [4, 21, 32, 33]. From a health-related selection perspective, it is important to take into account common early psychological risk factors of attention problems, externalizing behaviour, and low educational attainment, such as low IQ, as well as genetic risk factors [10, 27, 34–36]. These risk factors are not always measured (e.g. in the case of genetics), but can still partly be adjusted for by using statistical models that allow for the decomposition of variance into within- and between-person components [37, 38].

In order to evaluate social causation and health-related selection explanations in relation to the association between adolescents’ educational level and attention problems and externalizing behaviour, it is necessary to investigate whether educational level more strongly predicts these problem behaviours (i.e. social causation), or vice versa (i.e. health-related selection). This can be done using longitudinal datasets and cross-lagged panel models (CLPMs). To the best of our knowledge, all previous studies addressing this issue used either some form of cognitive testing or Grade Point Average (GPA)-based measures to assess educational performance [8, 39–51], and most were conducted in comprehensive educational systems, which are characterized by a late differentiation into educational tracks (e.g. in the USA at around age 18 upon completing high school) [8, 39, 41–44, 46, 47, 49–51]. This late differentiation means that unlike in the Dutch system adolescents’ educational level (as indicator of developing SES) does not become entrenched until the transition out of secondary school. It may therefore be quite difficult to assess adolescents’ developing SES in comprehensive educational systems, and GPA-based measures may indeed be the best indication in such systems. We identified only two studies from selective educational systems. These studies also used GPA-based measures, but did not take into account the role of adolescents’ educational tracks, which characterise selective educational systems [45, 48]. In selective educational systems, GPA-based measures only express performance relative to other students within the same track and are therefore not appropriate for predicting adolescents’ socioeconomic prospects.

Furthermore, many existing studies focus on specific developmental periods (e.g. early adolescence) [8, 39, 41, 45, 47, 49, 51]. However, it is pivotal to consider all phases of adolescent development simultaneously, as the importance of social causation and health-related selection mechanisms may differ across age groups. In addition, attention problems, externalizing behaviour, and academic outcomes may form developmental cascades [39]. For example, in a US study, attention problems predicted lower academic achievement in adolescents, which in turn predicted increased delinquent behaviour [39]. It is important to account for these cascading effects across phases of development, to strengthen causal inference.

Past studies based on GPA-based measures or cognitive tests have found different results for attention problems and externalizing behaviour [8]. When considering externalizing behaviour, four studies found mainly evidence for the social causation hypothesis [8, 39–41], with lower academic achievement predicting more problem behaviours, whilst three found mainly evidence for the health-related selection hypothesis, with externalizing behaviour predicting decreases in subsequent academic achievement [42–44]. Bidirectional associations between academic achievement and externalizing behaviours were found in seven studies [45–51]. In contrast, when considering attention problems, studies mainly support the health-related selection hypothesis, with symptoms more strongly predicting academic achievement than vice versa in two studies [8, 39]. These results underline the importance of making a distinction between attention problems and externalizing behaviour for answering our research questions rather than combining these concepts into one underlying dimension.

### Aims of the study

In the present study, we aimed to contribute to the literature by modelling reciprocal relationships (i.e. social causation and health-related selection) between attention problems and externalizing behaviour and adolescent educational level over a period of 16 years, using educational track membership as proxy for developing socioeconomic status (SES) in a selective educational system. Using educational track membership in the Dutch system as measure for educational attainment allows for assessing social mobility in adolescents at an earlier age than possible in comprehensive educational systems, as social stratification already occurs around the beginning of adolescence. Furthermore, we aimed to evaluate the role of family socioeconomic status (SES) and childhood IQ both as predictors of educational level, attention problems, and externalizing behaviour around age 14, and as confounders (‘third variables’) in cross-lagged paths. Finally, we address unmeasured time-stable confounding in cross-lagged paths using fixed effects.

## Materials and methods

### Study population

We used data from the first six waves (T1–T6) of the TRacking Adolescents’ Individual Lives Survey (TRAILS), a population-based prospective cohort study of Dutch adolescents. A detailed description of the cohort can be obtained elsewhere [52]. At the beginning of the study, 135 schools in the province of Groningen were invited, of which 122 decided to participate [53]. Adolescents were followed between 2000 and 2017 with assessments around age 11 (*n* = 2229), 14 (*n* = 2148), 16 (*n* = 1818), 19 (*n* = 1880), 22 (*n* = 1781), and 26 (*n* = 1616). Attrition analyses (Table S1) revealed that male gender, non-Dutch ethnicity, lower educational level, IQ, and parental SES were associated with dropout. Higher externalizing behaviour also predicted dropout, but only at wave 3 and 4. Attention problems were not associated with attrition. Some similar differences were found when comparing participants with complete information on educational level to those whose educational level was missing or could not be classified (Table S2). To deal with missing information, full-information maximum likelihood (FIML) was implemented, allowing to incorporate information from all participants.

### Adolescents’ educational level

**Fig. 1:**
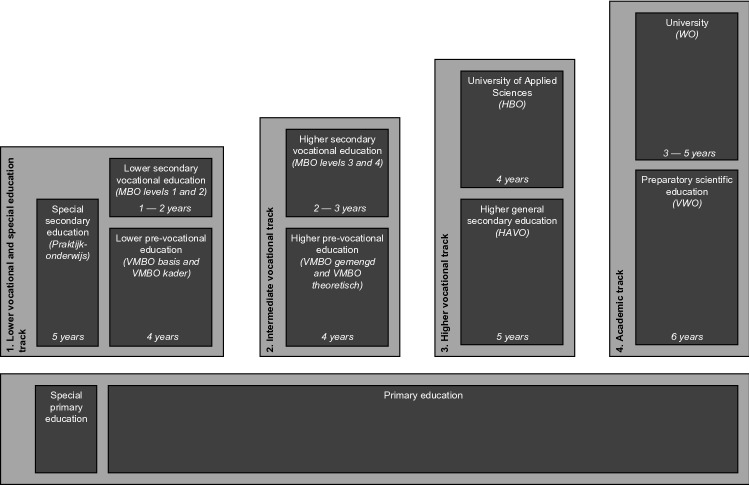
The Dutch educational system

The Dutch educational system is characterized by an early (age 11–12) selection into a particular educational track, based on cognitive tests and the advice of the primary school. There are four tracks in the Dutch educational system, each consisting of a specific type of secondary school followed by tertiary education at the corresponding level (Fig. 1): (1) lower vocational track, (2) intermediate vocational track, (3) higher vocational track, (4) academic track. In addition, there is a special education track, attended by students who are unable to attend regular education. This track was collapsed with the lower vocational track in our analyses. While in secondary education, adolescents can be recommended by their school to move between educational tracks, depending on their academic performance. Furthermore, after attaining specific milestones of their track, students can become eligible to continue their education in the next higher track. For example, students who finish the intermediate vocational track with an MBO level 4 diploma may continue their education by attending a University of Applied Sciences of the higher vocational track [54]. Overall, a substantial proportion of students is mobile between educational tracks: 24.66% of adolescents moved to a different track between wave 2 and 3, 25.41% between wave 3 and 4, 26.79% between wave 4 and 5, and 12.98% between wave 5 and 6, respectively. 47.00% of TRAILS participants were in a different educational track around age 26 (wave 6) than around age 14 (wave 2). Educational track membership was assessed at each wave by asking for participants’ current enrolment, as well as their highest completed diploma. Participants who finished the final diploma of a given track received the value corresponding to that level for all subsequent waves, unless they continued education at a higher level. Our measure of educational level allows us to assign a score that represents an age-appropriate measure of educational attainment as proxy of developing SES. Missing information on educational track membership from waves 2 through 6 was imputed using retrospective event history calendars conducted at wave 3 and wave 5. Participants who were still in elementary education or in a combined class at wave 2 were assigned according to their elementary school teachers’ recommended level. If this information was not available, pupils were classified according to the first track they attended after leaving elementary education or the combined class. It was not possible to classify participants who had not been in education for a longer period, were not classifiable into an educational track (e.g. because of education abroad), whose educational level was assessed incompletely, who did not respond to questions on education, or who had left the educational system permanently (wave 2: *N* = 221, 10.29%; wave 3: *N* = 289, 15.90%; wave 4: *N* = 373, 19.84%; wave 5: *N* = 352, 19.76%; wave 6 = 424, 26.24%). Educational level was set to missing for these participants.

### Adolescents’ mental health

Attention problems were assessed by calculating the mean scores (possible range 0–2) of the attention problem subscales of the Achenbach System of Empirically Based Assessment (ASEBA) Youth Self-report (YSR) (waves 2–3; 8 items) and Adult Self-report (ASR) (waves 4–6; 15 items) [55, 56]. The YSR and ASR contain lists of questions on emotional and behavioural problems in the preceding 6 months, with three response categories: 0, ‘not true’; 1, ‘somewhat or sometimes true’; 2, ‘very or often true’ [15]. The item ‘my schoolwork is bad’ was removed from the YSR attention problem scale, as it reflects the main mechanism through which attention problems affect educational level rather than attention problems themselves. Sample items of the attention problem scale are ‘I have difficulties concentrating’ and ‘I don’t finish tasks I begin’. Cronbach’s alphas for the attention problem scales ranged from 0.71 to 0.73 for the YSR, and from 0.84 to 0.86 for the ASR.

Externalizing behaviour was assessed by taking the mean score (possible range 0–2) of the externalizing subscales of the YSR (waves 2–3; 29 items) and ASR (waves 4–6; 33 items) [55, 56]. Items on substance use were removed from the scales (3 for the YSR and 2 for the ASR). This decision was made because adolescents’ substance use is heavily influenced by cultural factors and often shows unique relationships with educational level, which diverge from other externalizing behaviours [57]. For example, increased alcohol use has been associated with lower educational level in early adolescence, and higher educational level in late adolescence and young adulthood [58, 59]. Sample items from the externalizing behaviour scales are ‘I steal’ and ‘I physically attack others’. Cronbach’s alphas for the YSR ranged from 0.85 to 0.86, and for the ASR from 0.87 to 0.89.

### Characteristics at baseline (wave 1)

Demographic baseline characteristics were adolescent age, gender, area of residence (City of Groningen, Leeuwarden, Assen, other regions), and ethnicity. Children were classified as having non-Dutch ethnicity if at least one of their parents was born outside the Netherlands [60]. In addition, we included the following two baseline characteristics that were hypothesized to be associated with attention problems, externalizing behaviour, and education based on earlier studies [4, 5, 21, 27, 32, 33, 35, 36, 61, 62]:

1. *Parents’ socioeconomic position (SES)*, constructed as the mean score (observed range − 1.94 to 1.73) of the following five indicators (standardized): maternal and paternal educational attainment, maternal and paternal occupational position (according to the International Standard Classification of Occupations), and family income) [63].
2. *Intelligence Quotient (IQ)* was estimated using the Block Design and Vocabulary subtests of the Revised Wechsler Intelligence Scale for Children (WISC-R) (observed range 45–149) [64].

### Analytic approach

First, we computed descriptive statistics of the study population by cross-tabulating baseline characteristics (mean age 11) with early adolescent educational track membership at wave 2 (mean age 14), and attention problems and externalizing behaviour with concurrent educational level from wave 2 through wave 6 (mean age 26). Second, we conducted standardized linear regression models to estimate the relative importance of parental SES and adolescents’ IQ at baseline in predicting attention problems, externalizing behaviour, and educational level at wave 2. Third, we computed cross-lagged panel models (CLPMs) between attention problems and externalizing behaviour and educational level from wave 2 through wave 6. The CLPM estimates prospective associations between educational level and changes in subsequent attention problems and externalizing behaviour, and between attention problems and externalizing behaviour and changes in subsequent educational level, whilst taking into account temporal stability and reciprocity [37]. We first conducted separate CLPMs for attention problems and externalizing behaviour (see Fig. S1 for a schematic illustration), both unadjusted and adjusted for demographics, parental SES, and IQ. Subsequently, we combined attention problems and externalizing behaviour in a single CLPM to estimate their unique relationships with educational level (Fig. S2).

In addition, we conducted CLPMs with fixed effects, according to a specification by Allison et al. [37]. These models only use within-person variance to estimate associations between cross-lagged variables, hereby adjusting for all measured and unmeasured time-invariant characteristics. Separate one-sided fixed effects models were fit to assess lagged associations from education to changes in subsequent attention problems or externalizing behaviour, and from attention problems or externalizing behaviour to changes in subsequent education (Fig. S3). The fixed effects terms were represented by a latent variable of all measurements of the outcome with each having its factor loading constrained to be 1. This latent variable was allowed to be correlated freely with all time-varying exogenous variables in the model. Reciprocal causation was accommodated by including correlations between the error term of the outcome at each measurement occasion and all future values of the time-varying exposure [37]. Unlike in the traditional CLPM, we were not able to combine attention problems, externalizing behaviour, and education in a fixed effects model, when assessing the direction from education to attention problems/externalizing behaviour (i.e. social causation), as this led to convergence problems in Mplus (correlations > 1). We were able to compute such a fixed effects model, which additionally included baseline covariates, for the direction from attention problems/externalizing behaviour to education, with results identical to the fixed effects models assessing attention problems and externalizing behaviour seperately, and which did not include baseline covariates (Fig. S4). We hence assessed cross-lagged associations between externalizing behaviour and education, and attention problems and education, respectively, in separate fixed effects models without covariates (Fig. S3).

Model fit in Structural Equation Modelling (SEM) analyses was assessed using the Comparative Fit Index (CFI), the Tucker Lewis Index (TLI), the Root Mean Square Error of Approximation (RMSEA), and the Standardised Root Mean Residual (SRMR). Following the suggestions by Hu & Bentler, model fit was judged as ‘good’ if the CFI and TLI were > 0.95, the RMSEA was < 0.06, and the SRMR was < 0.08 [65]. Standard errors were estimated using robust maximum likelihood (MLR). Analyses were conducted in Mplus 8.6.

### Sensitivity analyses

In theory, fixed effects models require consistent measures of constructs over time [66]. In practice, this is often difficult to achieve in developmental research, which frequently aims to study multiple phases of development simultaneously. For example, oftentimes different, yet closely related, instruments are used to increase validity when assessing the same types of problem behaviours at different developmental stages. In TRAILS, the YSR is used to assess attention problems and externalizing behaviour in early and mid-adolescence, while the ASR is used in late adolescence and young adulthood. While it is common practice to combine YSR and ASR in the same models [57, 67, 68], we have conducted a sensitivity analysis of the fixed effects models using amended scales consisting only of corresponding items between the YSR and ASR (Table S3), to test whether using these slightly different instruments at different time points may have affected our findings**.**

Externalizing behaviour, attention problems, and educational problems frequently co-occur with internalizing problems [34, 48]. In order to study the unique associations between externalizing behaviour, attention problems, and education, some researchers have therefore suggested to additionally adjust for internalizing symptoms in CLPMs [48]. We have hence conducted a second sensitivity analysis in which the multivariate CLPM was additionally adjusted for the mean score of the summed anxious/depressed and withdrawn/depressed subscales of the YSR and ASR. Finally, we checked whether there were sex differences in cross-lagged associations in our fixed effects models, using multigroup modelling [69].

## Results

### Descriptive statistics

Table 1 shows the characteristics of TRAILS participants around age 11 according to educational level around age 14. Children with less affluent or non-Dutch parents more commonly attended the lower educational tracks. Girls more frequently attended the academic and intermediate vocational tracks than boys. Children in the lower vocational track and the academic track were slightly older at baseline than those in the intermediate and higher vocational tracks. Further, higher IQ around age 11 was associated with higher education around age 14.

**Table 1:** Characteristics of adolescents participating in the TRAILS Study (the Netherlands, 2000–2017, *N* = 2229) at wave 1 (2000–2002) according to educational level at wave 2 (2003–2005)

	All levels		Lower vocational & special education		Intermediate vocational		Higher vocational		Academic	
	*N* = 2229		*N* = 635		*N* = 497		*N* = 383		*N* = 457	
Male gender, *N* (%)	1098	(49.26)	341	(53.70)^a^	217	(43.66)^b^	196	(51.17)^a^	195	(42.67)^b^
Non-Dutch ethnicity, *N* (%)	301	(13.50)	108	(17.01)^a^	61	(12.27)^b^	39	(10.18)^b^	45	(9.85)^b^
Age, mean (SD)	11.11	(0.56)	11.16	(0.56)^a^	11.07	(0.54)^b^	11.05	(0.56)^b^	11.14	(0.56)^a^
Parental socioeconomic status (SES), mean (SD)	− 0.05	(0.80)	− 0.53	(0.70)^a^	− 0.16	(0.67)^b^	0.21	(0.68)^c^	0.55	(0.70)^d^
Wechsler Intelligence Devi-ation Quotient (IQ), mean (SD)	97.19	(15.00)	86.05	(12.49)^a^	95.20	(10.98)^b^	102.68	(11.20)^c^	111.14	(11.91)^d^

Parameters with different superscripts differ significantly from each other at *p *< 0.05, as determined by chi-squared tests (categorical variables) and one-way ANOVAs with pairwise comparisons (continuous variables)

*SD* standard deviation

Table 2 shows educational differences in attention problems and externalizing behaviour around ages 14, 16, 19, 22, and 26. Over the course of adolescence, educational differences in externalizing behaviour increased, with lower educational level being associated with more externalizing behaviour. When considering attention problems, adolescents in the academic track had lower scores than the lowest two tracks around age 14, and lower scores than all other tracks at 16, but we did not find clear differences among the other educational tracks. From age 19 onwards, no ordered relationships between educational level and attention problems were found anymore.

**Table 2:** Mental health characteristics of adolescents and young adults in the TRAILS study (wave 2–6; the Netherlands, 2000–2017,  *N* = 2229) according to concurrent educational level

	Wave 2		Wave 3		Wave 4		Wave 5		Wave 6	
	*N* = 2148		*N* = 1818		*N* = 1880		*N* = 1781		*N* = 1616	
Date range	2003–2005		2005–2008		2008–2010		2012–2014		2016–2017	
Age, mean (SD)	13.57	(0.53)	16.28	(0.71)	19.08	(0.60)	22.29	(0.65)	25.66	(0.60)
Male gender, *N* (%)	1054	(49.07)	867	(47.69)	898	(47.77)	843	(47.33)	735	(45.48)
Educational level, *N* (%)										
Lower vocational & special education	635	(32.20)	349	(22.83)	161	(10.68)	136	(9.52)	78	(6.54)
Intermediate vocational	497	(25.20)	405	(26.49)	498	(33.02)	354	(24.77)	273	(22.90)
Higher vocational	383	(19.42)	362	(23.68)	475	(31.50)	594	(41.57)	489	(41.02)
Academic	457	(23.17)	413	(27.01)	374	(24.80)	345	(24.14)	352	(29.53)
Mental health characteristics										
Attention problems, mean (SD)										
All levels	0.56	(0.34)	0.59	(0.35)	0.45	(0.32)	0.43	(0.32)	0.45	(0.34)
Lower vocational & special education	0.57	(0.34)^a^	0.64	(0.36)^a^	0.45	(0.34)^a^	0.44	(0.32)^a/b^	0.48	(0.33)^a^
Intermediate vocational	0.59	(0.34)^a^	0.61	(0.34)^a^	0.45	(0.33)^a^	0.40	(0.29)^a^	0.41	(0.29)^a^
Higher vocational	0.57	(0.34)^a/b^	0.61	(0.32)^a^	0.48	(0.31)^a^	0.45	(0.32)^b^	0.45	(0.33)^a^
Academic	0.53	(0.33)^b^	0.54	(0.34)^b^	0.44	(0.31)^a^	0.41	(0.34)^a/b^	0.43	(0.36)^a^
Externalizing problems, mean (SD)										
All levels	0.31	(0.21)	0.32	(0.21)	0.23	(0.21)	0.19	(0.18)	0.20	(0.19)
Lower vocational & special education	0.32	(0.21)^a^	0.37	(0.24)^a^	0.28	(0.26)^a^	0.22	(0.19)^a^	0.25	(0.21)^a^
Intermediate vocational	0.32	(0.21)^a^	0.34	(0.22)^a^	0.23	(0.21)^b^	0.20	(0.18)^a^	0.19	(0.18)^b/c^
Higher vocational	0.30	(0.20)^a/b^	0.31	(0.20)^b^	0.23	(0.20)^b^	0.19	(0.19)^a^	0.19	(0.18)^b^
Academic	0.28	(0.18)^b^	0.27	(0.18)^c^	0.19	(0.18)^c^	0.16	(0.16)^b^	0.17	(0.16)^c^

Parameters with different superscripts differ significantly from each other at *p* < 0.05, as determined by one-way ANOVAs with pairwise comparisons

*SD* standard deviation

Table 3 shows prospective associations between baseline (around age 11) characteristics and educational level, attention problems, and externalizing behaviour around age 14. In bivariate results, higher parental SES and child IQ strongly predicted higher educational level around age 14. These results remained robust after adjusting for covariates, be it that a relatively large proportion of the association between SES and education was explained by differences in IQ. Higher parental SES also predicted lower attention problems and externalizing behaviour, albeit to a lesser extent. Baseline IQ was neither associated with attention problems nor externalizing behaviour at around age 14.

**Table 3:** The association between baseline characteristics (wave 1) and educational level, externalizing behaviour, and attention problems at wave 2 in the TRAILS Study (the Netherlands, 2000–2017, *N* = 2229); unadjusted and adjusted linear regression models (stdyx-standardized *ß*-coefficient, robust standard error, *p*-value)

	Model 1	Model 2	Model 3
Educational level			
Parental socioeconomic status (SES)	**0.506 (0.017), *p* < 0.001**	**0.498 (0.018), *p* < 0.001**	**0.303 (0.018), *p* < 0.001**
Wechsler Intelligence Deviation Quotient (IQ)	**0.621 (0.013), *p* < 0.001**	**0.629 (0.013), *p* < 0.001**	**0.516 (0.016), *p* < 0.001**
Externalizing behaviour			
Parental socioeconomic status (SES)	**− 0.067 (0.022), *p* = 0.002**	**− 0.068 (0.023), *p* = 0.003**	**− 0.067 (0.025), *p* = 0.006**
Wechsler Intelligence Deviation Quotient (IQ)	− 0.029 (0.023), *p* = 0.215	− 0.028 (0.023), *p* = 0.239	− 0.002 (0.025), *p* = 0.924
Attention problems			
Parental socioeconomic status (SES)	**− 0.045 (0.022), *p* = 0.046**	**− 0.064 (0.023), *p* = 0.005**	**− 0.062 (0.025), *p* = 0.012**
Wechsler Intelligence Deviation Quotient (IQ)	− 0.023 (0.023), *p* = 0.311	− 0.028 (0.023), *p* = 0.219	− 0.005 (0.025), *p* = 0.833

Model 1: unadjusted models

Model 2: adjusted for age, sex, municipality, and ethnicity at baseline (wave 1)

Model 3: adjusted for age, sex, municipality, ethnicity, and adjusted for parental socioeconomic status or IQ at baseline (wave 1)

Boldface denotes statistical significance at *p* < 0.05

### Cross-lagged associations for attentions problems and education

**Fig. 2:**
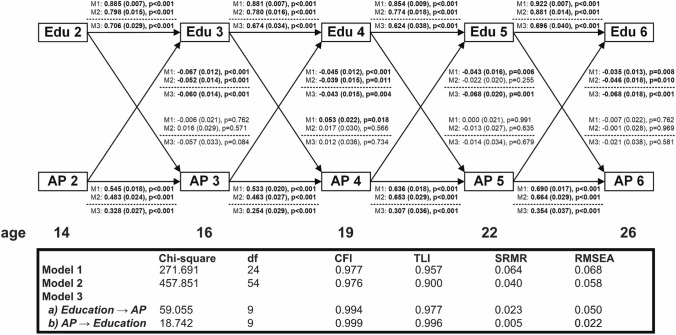
Bidirectional associations between educational level and attention problems in the TRAILS Study (the Netherlands, 2000–2017, *N* = 2229); linear regression coefficients (stdyx-standardized ß-coefficient, robust standard error, p-value) from cross-lagged panel models without (Model 1 and 2) and with fixed effects (Model 3). Model 1: bivariate cross-lagged panel model. Model 2: cross-lagged panel model adjusted for age, gender, area of residence, ethnicity, parental SES, and IQ at baseline (wave 1), and externalizing behaviour at each preceding wave. Model 3: cross-lagged panel models with fixed effects—adjustment for time-invariant characteristics was performed by inclusion of a latent variable. Edu, educational level; AP, attention problems. Boldface denotes statistical significance at *p* < 0.05

**Fig. 3:**
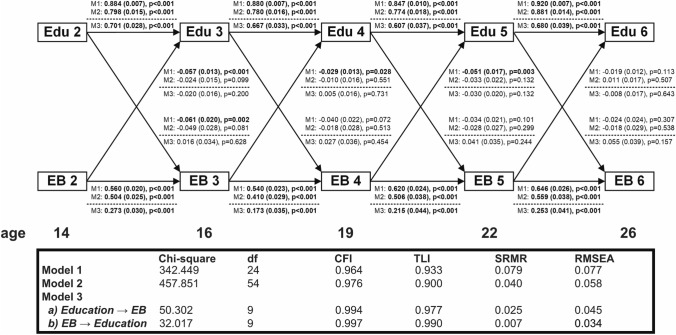
Bidirectional associations between educational level and externalizing behaviour in the TRAILS Study (the Netherlands, 2000–2017, *N* = 2229); linear regression coefficients (stdyx-standardized ß-coefficient, robust standard error, *p*-value) from cross-lagged panel models without (Model 1 and 2) and with fixed effects (Model 3). Model 1: bivariate cross-lagged panel model. Model 2: cross-lagged panel model adjusted for age, gender, area of residence, ethnicity, parental SES, and IQ at baseline (wave 1), and attention problems at each preceding wave. Model 3: cross-lagged panel models with fixed effects—adjustment for time-invariant characteristics was performed by inclusion of a latent variable. Edu, educational level; EB, externalizing behaviour. Boldface denotes statistical significance at *p* < 0.05

In bivariate CLPMs, educational level exhibited consistently high stability (standardized *ß* > 0.800; Figs. 2, 3). The stability of attention problems was also high and increased from a standardized *ß* of 0.545 in early adolescence to 0.690 in young adulthood (Fig. 2). Bivariate analyses showed that attention problems robustly predicted subsequent decreases in education throughout the entire study period. The same results were found in the fixed effects model, suggesting that these associations were not explained by time-invariant confounders. These associations also remained significant in the covariate-adjusted models (i.e. adjusted for demographics, parental SES, IQ, and concurrent externalizing behaviour), except for the path between age 19 and 22, which was no longer significant after adjusting for externalizing behaviour (Fig. S5).

When considering social causation, we only found one bivariate association between higher education around age 16 and increases in attention problems around age 19. This association was no longer significant after adjusting for parental SES, and disappeared almost completely after further taking into account adolescents’ IQ (Fig. S5). We found no significant associations between education and subsequent changes in attention problems in the fixed effects model.

The fixed effects models with the amended scales (Fig. S7) were in line with the main results, with significant paths from attention problems to subsequent decreases in educational level throughout the entire followup, and no siginificant paths from educational level to subsequent changes in attention problems. Also, further adjusting CLPMs for anxiety/depression did not change cross-lagged assocations between attention problems and education (Fig. S5). While we were not able to find significant differences in any cross-lagged associations between boys and girls in fixed effects models, we noted substantial increases in standard errors in the multigroup models, particularly regarding the social causation paths (Fig. S9). Furthermore, allowing for sex differences in cross-lagged paths did not improve model fit, as compared to models where cross-lagged paths were constrained to be equal across sexes.

### Cross-lagged associations for externalizing behaviour and education

Externalizing behaviour showed similar stability to attention problems, with slight increases over time, ranging from a standardized *ß* of 0.560 in early adolescence to 0.646 in young adulthood, in bivariate models (Fig. 3). When considering health-related selection, we found that externalizing behaviour predicted decreases in education from around age 14 through 22, but not from around age 22–26. These associations remained robust after adjusting for baseline covariates, but lost significance after additionally taking into account attention problems (Fig. S6). When considering social causation, we only found a significant association in early adolescence, with higher education around 14 predicting decreases in externalizing behaviour around 16. However, after adjusting for parental SES (Fig. S6), this association was no longer significant. We did not find any cross-lagged associations between education and externalizing behaviour in fixed effects models when using the unamended YSR/ASR scales.

Our sensitivity analyses showed very similar results, with two exceptions: first, a path from externalizing behaviour to decreases in subsequent education from around age 19 to around age 22 became borderline significant (*p* = 0.049) in the fixed effects model with amended scales (Fig. S8). Second, additional adjustment for anxiety/depression made the path from externalizing behaviour around age 14 to decreases in education around age 16 significant in the multivariate-adjusted CLPM (Fig. S6). However, the change in coefficients was only minor. Similar to the results concerning attention problems, we did not find significant differences in cross-lagged paths between education and externalizing behaviour in males and females in fixed effects models, and allowing for sex differences in cross-lagged paths did not improve model fit (Fig. S10).

Model fit of all cross-lagged models was adequate to good [65]. Overall, the fixed effects CLPMs fit the data better than the CLPMs without fixed effects.

## Discussion

In this study, we aimed to evaluate the social causation and health-related selection hypotheses by modelling reciprocal relationships of externalizing behaviour and attention problems with educational level throughout adolescence and young adulthood in a selective educational system. Attention problems almost consistently predicted subsequent decreases in educational level throughout adolescence and into young adulthood (i.e. health-related selection). This result was robust to adjustment for covariates and fixed effects. We also found bivariate associations between externalizing behaviour and subsequent decreases in educational level, but these paths were no longer significant after adjusting for concurrent attention problems, and neither in the fixed effects model when using the unamended scales. When considering social causation, two time-specific associations between education and subsequent changes in attention problems or externalising problems were found, but these were explained by time-stable confounders. Surprisingly, there were only few cross-sectional differences in attention problems across educational tracks.

### Interpretation of findings

Our results show that attention problems are consistently associated with subsequent decreases in educational level (i.e. health-related selection) throughout adolescence and young adulthood. Our findings are consistent with previous studies that highlight the direct detrimental effects of attention problems on educational outcomes above and beyond other mental health problems [1, 15], was well as with previously published CLPMs on GPA-based measures and attention problems [8, 39]. We add to this literature by showing that health-related selection effects may extend beyond GPA and could be sufficiently consequential for adolescents to change to a lower degree programme in the Dutch selective educational system.

Attention problems were associated with health-related selection similarly throughout all phases of adolescence and young adulthood.

While symptoms associated with attention problems, such as impulsivity and hyperactivity, tend to decline with age, attention problems often persist into young adulthood [70]. This persistence, in combination with gradual increases in self-management demands, may explain why health-related selection effects hardly diminish over time [71]. With extensive teacher and parental support, some adolescents may be able to attain recommendations for the higher educational tracks in primary school despite high levels of attention problems. However, once in secondary school, many of these adolescents may struggle with the increased workload, eventually leading them to switch to a lower educational track. Other adolescents grappling with attention problems may nevertheless successfully finish the secondary school of one of the higher tracks, but run into difficulties upon entering higher education, which requires much more autonomy and self-directed learning [71]. Matters are further complicated for these young adults due to a steep increase in personal responsibilities after leaving the parental home, often combined with a loss of contact with youth mental health care institutions [71, 72].

Unexpectedly, we found only few cross-sectional differences in attention problems across educational tracks. Only around age 14 we found lower scores in the academic track, as compared to the lowest two tracks, and around age 16 compared to all other tracks. We did not find clear differences amongst the other tracks, suggesting that the cross-sectional association between education and attention problems might not be linear in early and mid-adolescence. We found no increase in cross-sectional differences in attention problems over time, which would be expected if there is persistent downward educational mobility related to attention problems. Instead, from age 19 onwards, we no longer found an ordered relationship between educational level and attention problems. One explanation for these results could be selective attrition or missingness. Indeed, attrition analyses revealed higher dropout of adolescents from the lower educational tracks. However, attention problems were not associated with attrition, and participants with incomplete information on educational level only reported more attention problems at wave 6. This suggests that attrition or missingness may be no sufficient explanation of the scarcity of cross-sectional associations between attention problems and education from late adolescence onwards. Instead, this finding could be related to lower academic demands in combination with an earlier career choice in the vocational tracks. The theoretically focussed work in the higher educational tracks may elicit more attention problems in adolescents than the more practical work in the vocational tracks. Furthermore, adolescents in the vocational tracks are able to choose their desired profession earlier, and can subsequently train in the job that suits their individual talents best. Taken together, this could have led to an equalization in cross-sectional educational differences in self-perceived attention problems.

Regarding social causation and attention problems, we found one bivariate path from higher education around age 16 to increases in attention problems around age 19, which was driven by adolescents’ IQ, and to a lesser extent parental SES. While attention problems have often been associated with lower IQ scores in the literature [35, 73], there is some evidence that individuals with high IQ may also be at increased risk of attention problems [74]. Similarly, higher parental SES is associated with both lower attention problems and higher educational attainment in the offspring [3, 5].

When considering health-related selection related to externalizing behaviour in bivariate CLPMs, we found significant associations of externalizing behaviour with subsequent decreases in education throughout all of adolescence and up to around age 22. These associations lost significance after additionally adjusting for attention problems and were absent in fixed effects models. It is therefore likely that decreases in educational level in adolescence are not predicted by adolescents’ delinquent or aggressive behaviour, but by co-occurring attention problems and associated traits, such as low effortful control, adversely affecting school performance [75–77].

We found a bivariate social causation path from lower educational level to increases in externalizing behaviour in early adolescence only, which was no longer significant after further adjusting for parental SES. The propensity of the Dutch educational system to place children from lower SES households in the lower educational tracks [5], in combination with the higher prevalence of stress factors predictive of externalizing behaviour in these families, such as neighbourhood disadvantage, stressful life events, and unhealthy family functioning [32], could thus partially explain how adolescents’ educational level and externalizing behaviour become associated.

The high stability of educational track membership over the course of adolescence and young adulthood highlights the importance of the transition from primary to secondary school around age 12 in explaining educational differences in adolescents’ attention problems and externalizing behaviour. Both parental SES and IQ strongly predicted adolescents’ educational level in early adolescence, which is in line with previous studies showing that these characteristics are amongst the strongest determinants of adolescents’ educational attainment [5, 62]. Low parental SES, but not IQ, predicted increased externalizing behaviour and attention problems around age 14, highlighting the importance of parental SES in adolescents’ problem behaviours [4, 33], as explained above.

### Limitations and strengths

Some limitations of this study may have affected our results and conclusions. First, we chose to operationalize adolescents’ attention problems and externalizing behaviour using the empirically-based syndrome scales of the YSR/ASR, which are less closely related to diagnosable conditions than the DSM-based clinical scales. This choice was made because the empirically based scales are more comparable in the YSR and the ASR than the DSM-based scales. Different diagnoses (and hence different DSM-based scales) are used to represent externalizing behaviour in adolescents (i.e. oppositional defiant disorder and conduct disorder) and adults (i.e. antisocial personality disorder). Likewise, in adolescents with ADHD, symptoms of impulsivity and hyperactivity often diminish over time, while attention problems tend to remain more stable and often persist into adulthood [70]. Moreover, previous research on ADHD patients has shown that attention problems are more predictive of educational outcomes than impulsivity and hyperactivity [23]. That said, the empirically based YSR and ASR scales are strongly correlated with the DSM-oriented scales of related conditions. Achenbach et al., reported correlations of the attention problems scale with the ADHD scale of *r* = 0.91 for the ASR [78], and a Cohen’s kappa of 0.70 for the correlation between scoring in the borderline/clinical range of the attention problems and the ADHD scale of the YSR [79]. Although the empirically based scales of the YSR and ASR were far more comparable than the DSM-based scales, still slightly different questionnaires were used to capture attention problems and externalizing behaviour in adolescents and young adults, in order to account for developmental differences. While it is commonplace in the literature to combine these scales in longitudinal models of development [57, 67, 68], we have conducted sensitivity analyses to rule out the possibility that this affected the outcomes of the fixed effects models [66]. Overall, these analyses (which used only concordant items between the YSR and ASR) yielded similar results as the main analyses.

Second, attrition might have influenced the results of our study. Although we implemented FIML to manage missing data, higher dropout of adolescents with less favourable conditions (e.g. lower education, parental SES, IQ) may still have affected our results. As these characteristics are also important determinants of adverse outcomes in young adulthood, further research on at-risk groups is necessary [80]. Third, while we have used fixed effects to address unmeasured time-invariant confounding, residual confounding may still have affected our results. For example, our fixed effects models did not take into account that unmeasured time-stable characteristics (e.g. genetics) may have time-varying effects. Further residual confounding may stem from other time-varying variables we have not measured. Fourth, while we did not find significant differences in cross-lagged associations between genders, we found increased standard errors in the multigroup models, suggesting that we might need larger samples to detect smaller differences between groups. Further studies with larger samples are needed to investigate variations in cross-lagged associations by gender, parental SES, and ethnicity.

Our study has several key strengths. First, the TRAILS Study is characterized by a long follow-up (16 years) and a high response rate [52]. Second, by using innovative statistical techniques we simultaneously took into account reciprocity between educational level and mental health, disentangled their temporal direction, and addressed time-invariant unmeasured confounding using fixed effects [37]. Third, we add to the literature by, for the first time, modelling bidirectional associations between educational level and externalizing problems in a selective educational system, which provides a consistent and age-appropriate measure of educational attainment, as proxy for developing SES over the course of adolescence. The selection into educational tracks as early as at age 11–12 years means that Dutch adolescents grow up in distinct educational environments that are characterized by different social norms, future expectations, cognitive resources, and occupational prospects [7, 18]—characteristics that are closely related to conceptualizations of SES in adulthood [20]. One could therefore argue that in selective educational systems, such as in the Netherlands, youngsters move into ‘their own’ SES at a much earlier age than in comprehensive systems, such as in Finland or the USA. TRAILS provides a unique opportunity to investigate both the antecedents and consequences, in terms of health-related characteristics, of this differentiation and subsequent intragenerational social mobility in adolescents and young adults.

### Conclusion and implications

In line with the health-related selection hypothesis, our findings suggest that attention problems pose a risk for decreases in educational attainment during all phases of adolescence and young adulthood, as youngsters with these kinds of problems may face substantial academic difficulties in the higher educational tracks. Although the cross-lagged paths as such may represent small effects, their cumulative effect throughout adolescence may be considerable. Our results emphasize the need for interventions to address the negative impact of attention problems on educational attainment. There is some evidence that pharmacological treatment [81–83] and certain non-pharmacological interventions [84, 85] may provide short-term improvements in GPA. However, only few studies have investigated to what extent these improvements are sufficient to meaningfully alter adolescents’ and young adults’ educational careers [83, 86]. Reassuringly, a registry-based study has found that ADHD treatment was associated with a decreased risk of non-eligibility to upper secondary school in Sweden [22]. Other studies have concluded that treatment effects may not be strong enough to address disparities in educational attainment [83, 86, 87]. Future research on interventions should focus on their long-term effectiveness, effect sizes, and clinically meaningful indicators [83, 86], such as long-term educational attainment.

Regarding social causation, differences in parents’ SES across educational tracks seem to contribute to educational differences in adolescents’ externalizing behaviour in mid-adolescence. Interventions targeting externalizing behaviours should therefore take into account risk factors related to low family SES, such as lack of economic resources, unhealthy family functioning, stressful life events, and neighbourhood disadvantage [32].

## Supplementary Information

Below is the link to the electronic supplementary material.

Supplementary file1 (DOCX 5848 kb)

## Data Availability

Under the General Data Protection Regulation (GDPR), our dataset is considered pseudonymized rather than anonymized, and is still regarded as personal data. When participants were invited to the cohort more than 20 years ago, they were not asked to give informed consent to make their personal data publicly available in pseudonymized form. As a result of this, legal and ethical restrictions prevent the authors from making data from the TRAILS Study publicly available. Data are available upon request from the TRAILS data manager (trails@umcg.nl). Detailed information about the participation agreements with TRAILS participants is available from the ethics committee; Central Committee on Research Involving Human subjects (CCMO; tc@ccmo.nl). For more information about accessing data from the TRAILS Study, please see https://www.trails.nl/en/hoofdmenu/data/data-use. The Mplus syntax for our analyses can be obtained from: https://github.com/hschmengler/Educational-level-attention-problems-and-externalizing-behaviour-in-adolescence-and-early-adulthood
