# Supplementary material for: Educational level, attention problems, and externalizing behaviour in adolescence and early adulthood: the role of social causation and health-related selection—the TRAILS study
Source: Eur Child Adolesc Psychiatry. 2021 Nov 19;32(5):809–24. doi: 10.1007/s00787-021-01913-4 (PMC10147770; doi:10.1007/s00787-021-01913-4)
Supplement: Supplementary file 1 — Supplementary file1 (DOCX 5848 kb) [file 787_2021_1913_MOESM1_ESM.docx]

**ONLINE SUPPLEMENT**

**Fig. S1** Path diagram of a bivariate cross-lagged panel model

*Edu = educational level; AP = attention problems; EB = externalizing behaviour.*

**Fig. S2** Path diagram of a cross-lagged panel model with multiple time-varying variables, adjusted for time-invariant baseline characteristics

*Baseline (wave 1) covariates include age, gender, area of residence, ethnicity, parental SES, and IQ and predict all time-varying variables (i.e. education, attention problems, externalizing behaviour) at all subsequent waves. Arrows from wave 1 covariates pointing into the time-varying variables are not displayed in the figure for reasons of clarity.*

*Edu = educational level; AP = attention problems; EB = externalizing behaviour.*

**Fig. S3** Path diagrams of one-sided cross-lagged panel models with fixed effects (without covariates) according to the specification by Allison et al. [1]; separate fixed effects models were fit to assess each of the two hypothesized causal directions between educational level and attention problems (AP) and externalizing behaviour (EB) *Edu = educational level; AP = attention problems; EB = externalizing behaviour.*

**Fig. S4** Covariate-adjusted one-sided cross-lagged panel model with fixed effects for the direction from attention problems/externalizing behaviour (AP/EB) to educational level (i.e. health-related selection) in the TRAILS Study (the Netherlands, 2000–2017, N = 2,229); linear regression coefficients (stdyx-standardized ß-coefficient, robust standard error, p-value)


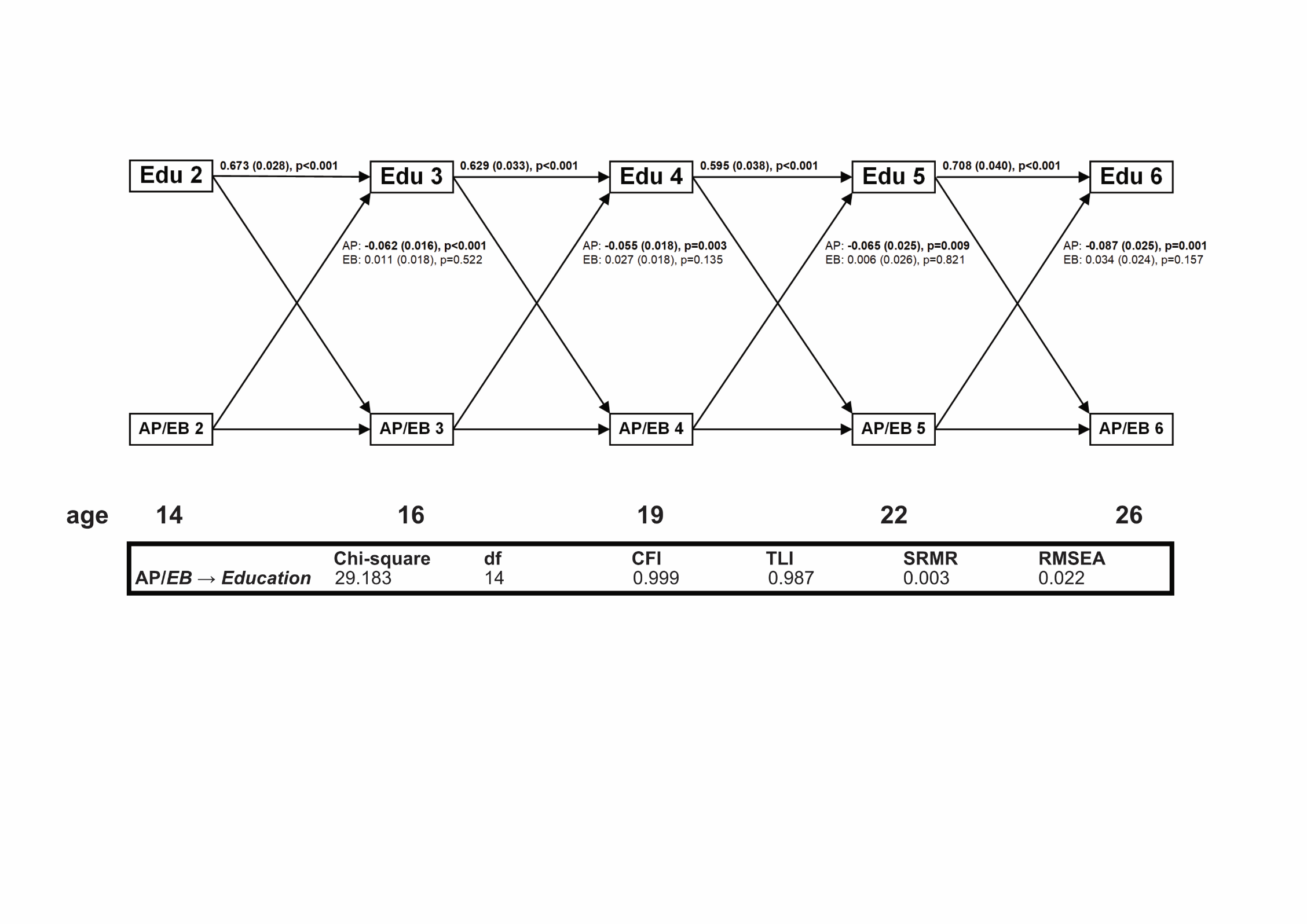


*Adjusted for baseline (wave 1) covariates (i.e. age, gender, area of residence, ethnicity, parental SES, and IQ). Attention problems and externalizing behaviour are mutually adjusted. Adjustment for unassessed time-invariant characteristics was performed by inclusion of a latent variable.*

*Edu = educational level; AP = attention problems; EB = externalizing behaviour.*

***Boldface*** *denotes statistical significance at p < 0.05.*

**
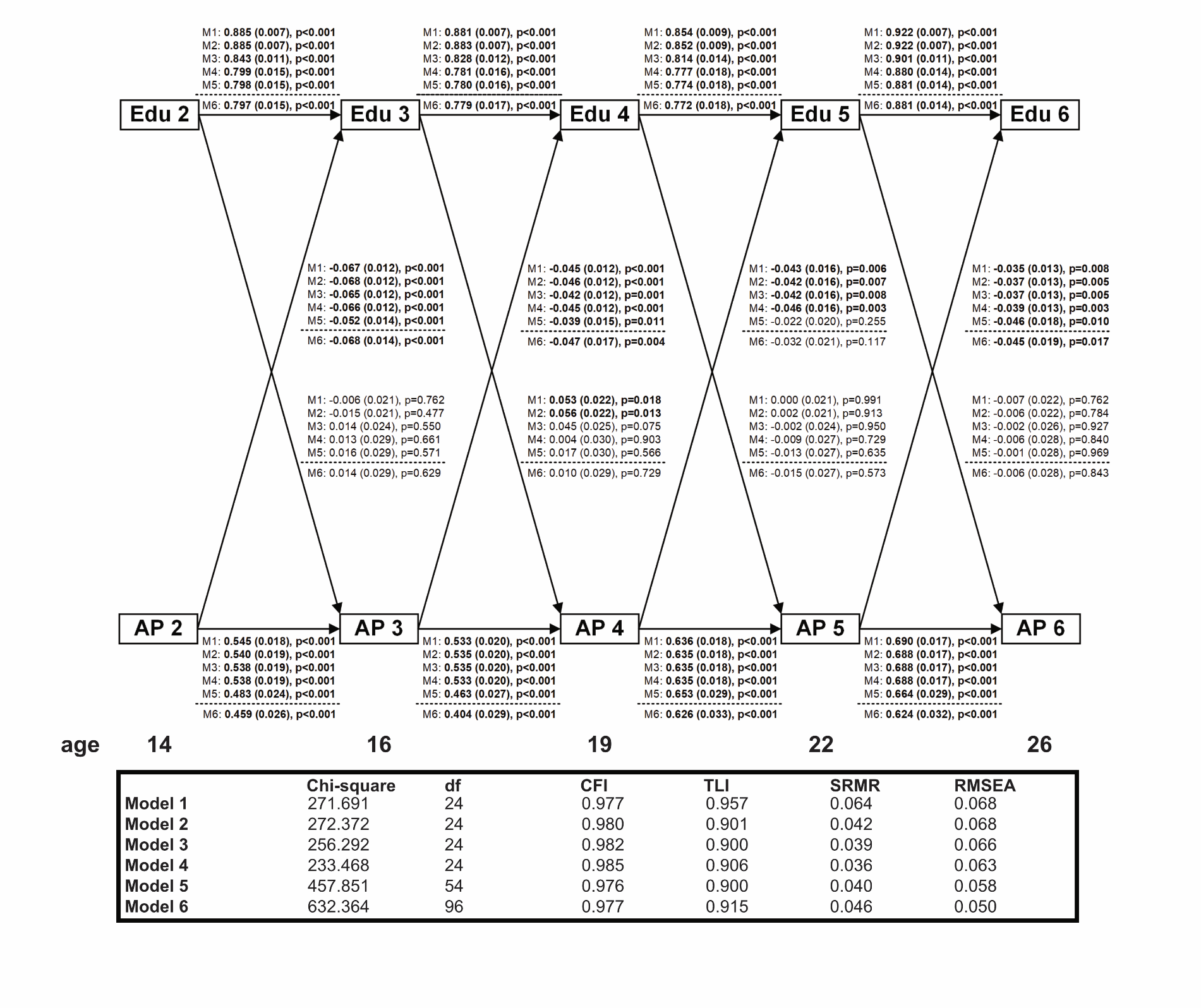
Fig. S5** Bidirectional associations between educational level and attention problems (AP) in the TRAILS Study (the Netherlands, 2000–2017, N = 2,229); sequentially adjusted linear regression coefficients (stdyx-standardized ß-coefficient, robust standard error, p-value) for different sets of covariates

*Model 1: bivariate cross-lagged panel model.*

*Model 2: cross-lagged panel model adjusted for demographics (age, gender, area of residence, and ethnicity) at baseline (wave 1).*

*Model 3: cross-lagged panel model adjusted for demographics and parental SES at baseline (wave 1).*

*Model 4: cross-lagged panel model adjusted for demographics, parental SES, and IQ at baseline (wave 1).*

*Model 5: cross-lagged panel model adjusted for demographics, parental SES, and IQ at baseline (wave 1), and externalizing behaviour at each preceding wave.*

*Model 6: cross-lagged panel model adjusted for demographics, parental SES, and IQ at baseline (wave 1), and externalizing behaviour and depression/anxiety at each preceding wave.*

*Edu = educational level; AP = attention problems.*

***Boldface*** *denotes statistical significance at p < 0.05.*

**
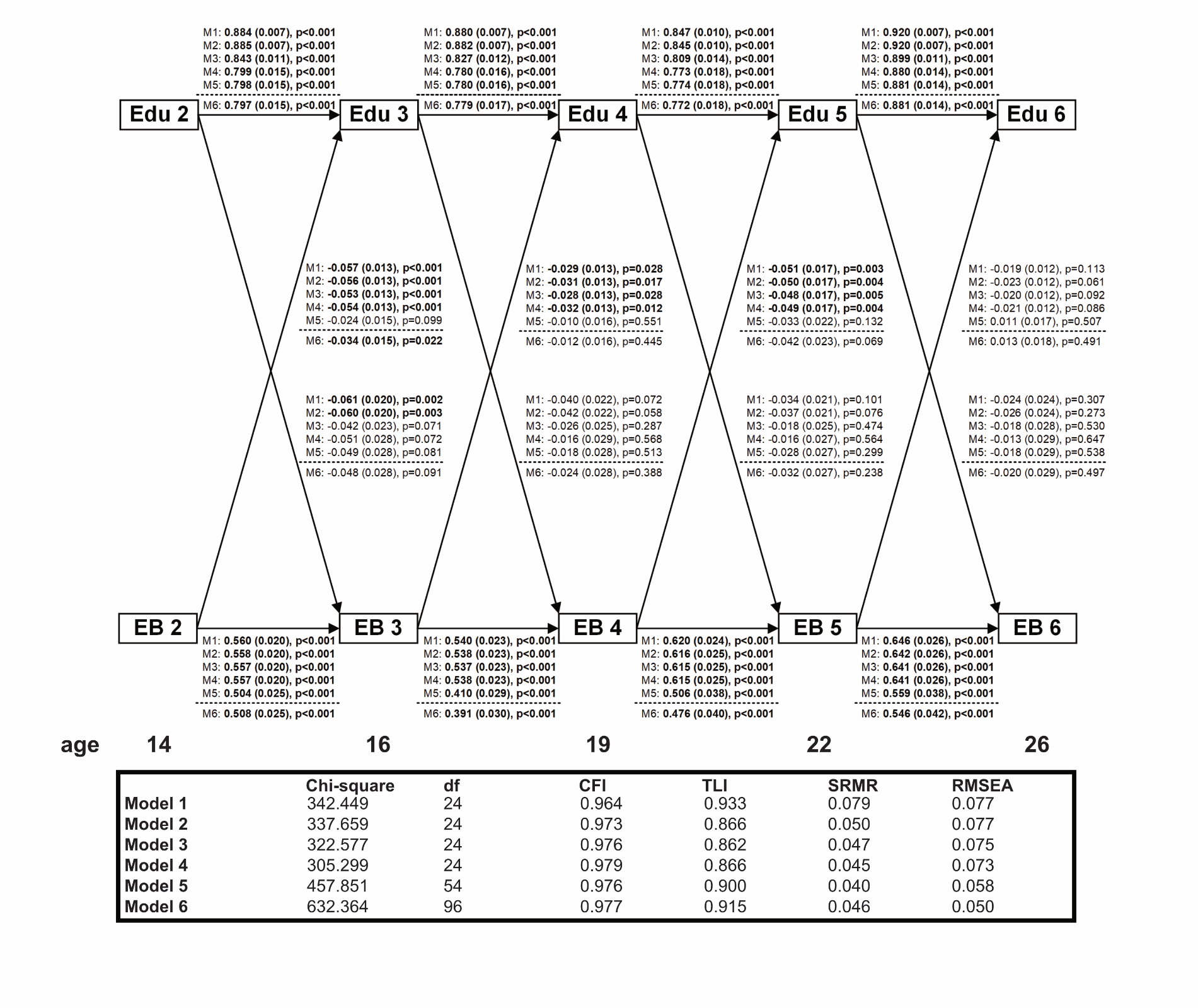
Fig. S6** Bidirectional associations between educational level and externalizing behaviour (EB) in the TRAILS Study (the Netherlands, 2000–2017, N = 2,229); sequentially adjusted linear regression coefficients (stdyx-standardized ß-coefficient, robust standard error, p-value) for different sets of covariates

*Model 1: bivariate cross-lagged panel model.*

*Model 2: cross-lagged panel model adjusted for demographics (age, gender, area of residence, and ethnicity) at baseline (wave 1).*

*Model 3: cross-lagged panel model adjusted for demographics and parental SES at baseline (wave 1).*

*Model 4: cross-lagged panel model adjusted for demographics, parental SES, and IQ at baseline (wave 1).*

*Model 5: cross-lagged panel model adjusted for demographics, parental SES, and IQ at baseline (wave 1), and attention problems at each preceding wave.*

*Model 6: cross-lagged panel model adjusted for demographics, parental SES, and IQ at baseline (wave 1), and attention problems and depression/anxiety at each preceding wave.*

*Edu = educational level; EB = externalizing behaviour.*

***Boldface*** *denotes statistical significance at p < 0.05.*

**Fig. S7** Bidirectional associations between educational level and attention problems (AP), using amended scales, in the TRAILS Study (the Netherlands, 2000–2017, N = 2,229); linear regression coefficients (stdyx-standardized ß-coefficient, robust standard error, p-value) from cross-lagged panel models with fixed effects

*
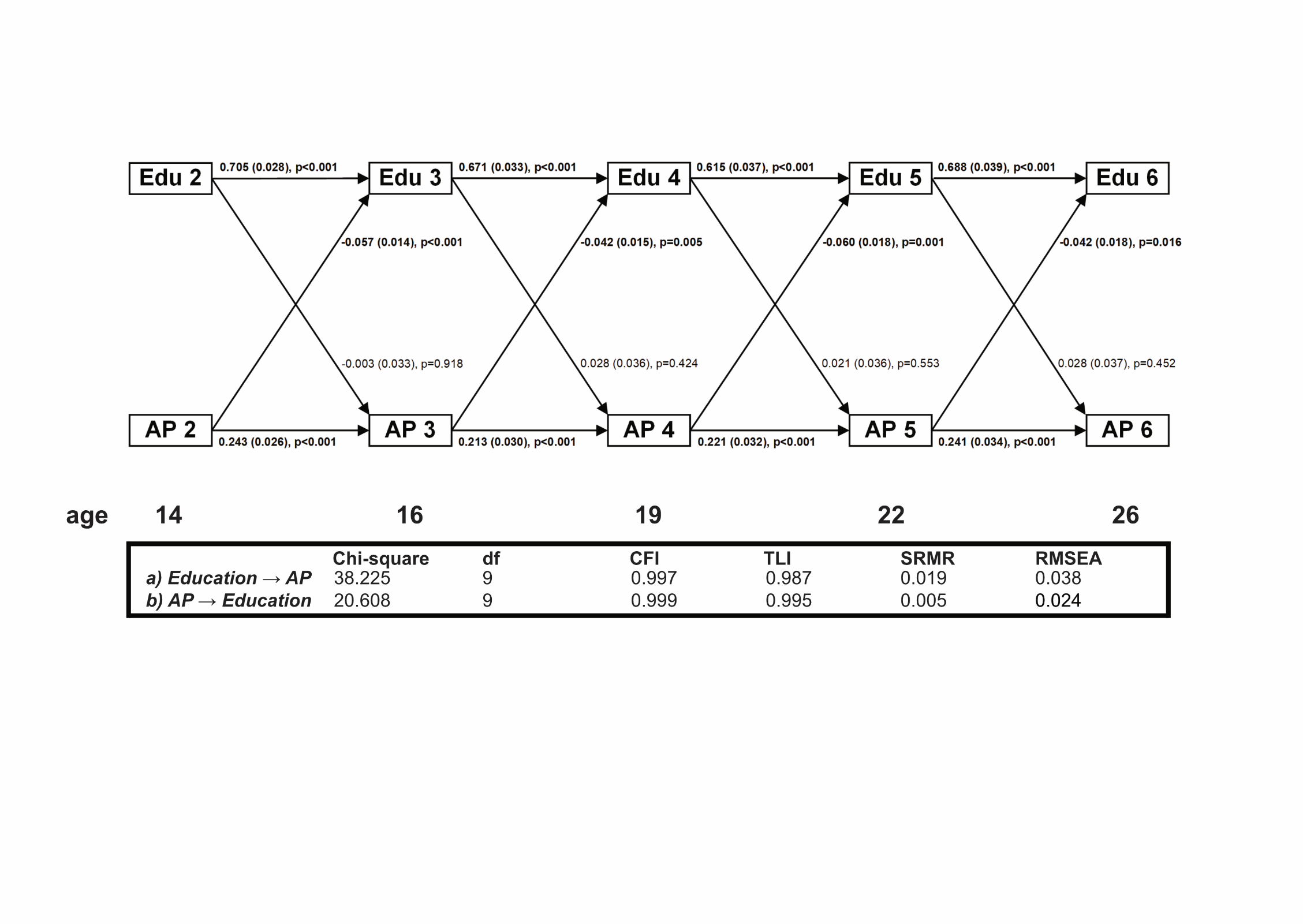
*

*Adjustment for time-invariant characteristics was performed by inclusion of a latent variable.*

*Edu = educational level; AP = attention problems.*

***Boldface*** *denotes statistical significance at p < 0.05.*

**Fig. S8** Bidirectional associations between educational level and externalizing behaviour (EB), using amended scales, in the TRAILS Study (the Netherlands, 2000–2017, N = 2,229); linear regression coefficients (stdyx-standardized ß-coefficient, robust standard error, p-value) from cross-lagged panel models with fixed effects

**
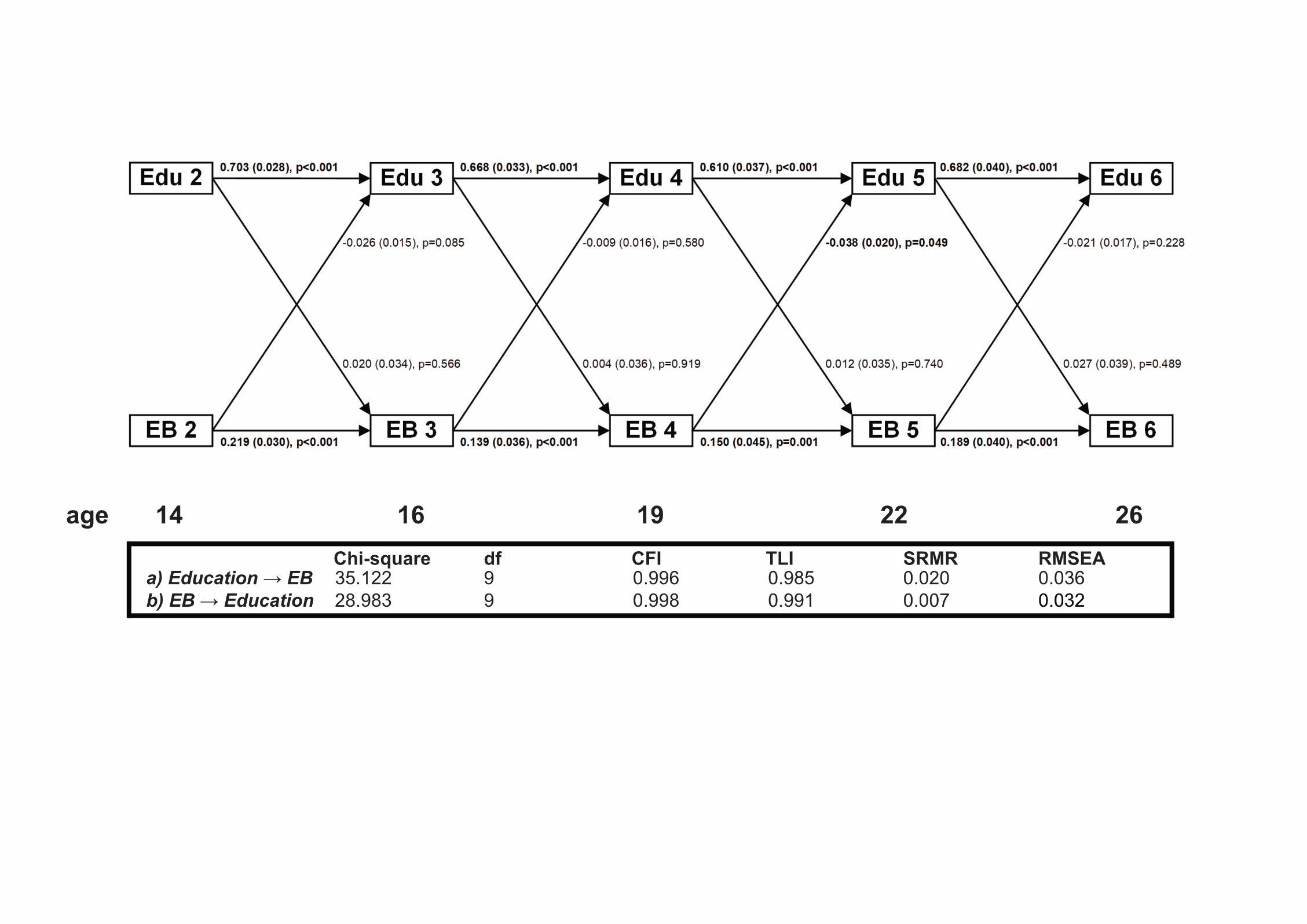
**

*Adjustment for time-invariant characteristics was performed by inclusion of a latent variable.*

*Edu = educational level; EB = externalizing behaviour.*

***Boldface*** *denotes statistical significance at p < 0.05.*

**Fig. S9** Bidirectional associations between educational level and attention problems (AP) in males and females, in the TRAILS Study (the Netherlands, 2000–2017, N = 2,229); linear regression coefficients (stdyx-standardized ß-coefficient, robust standard error, p-value) from cross-lagged panel models with fixed effects

**
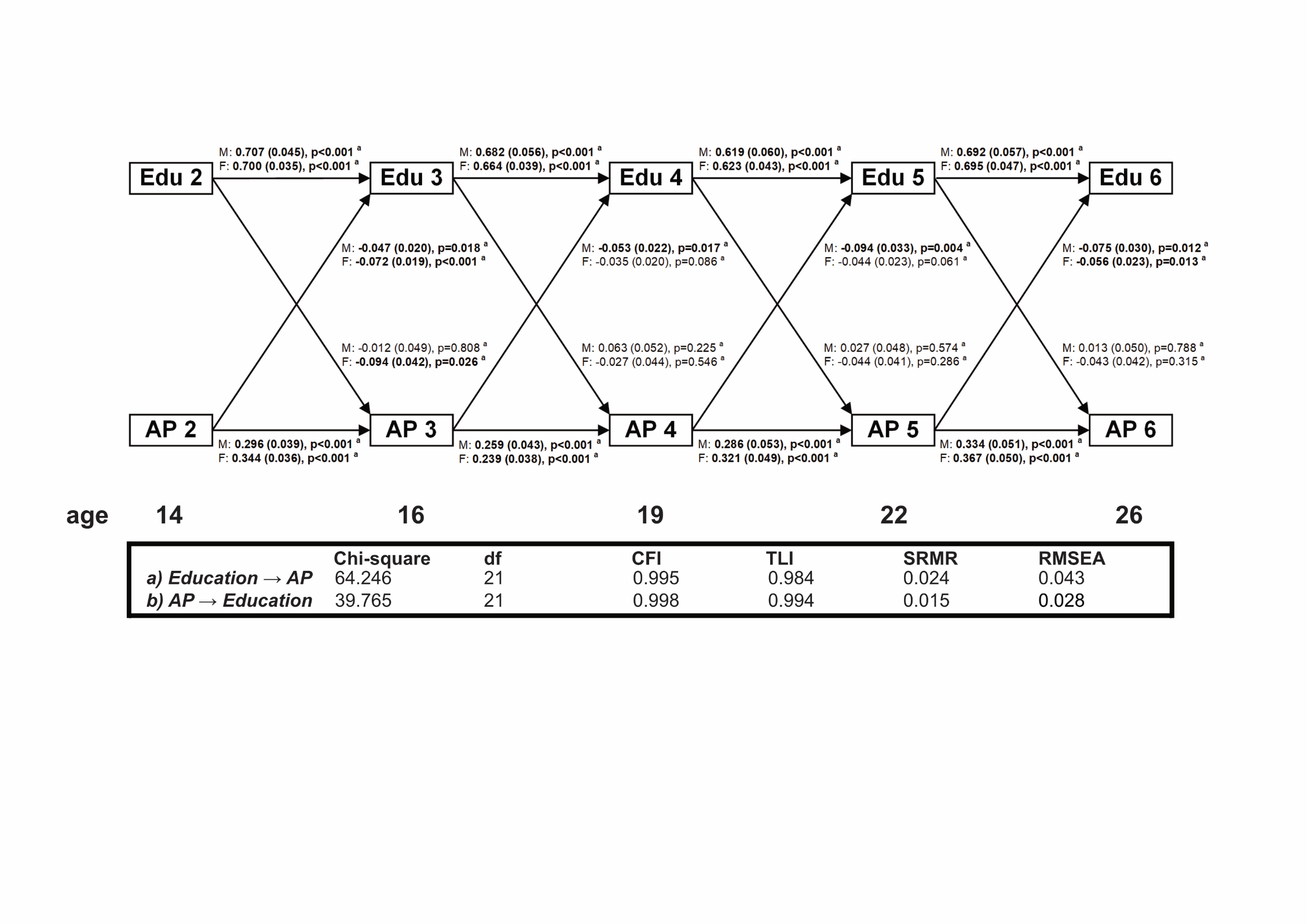
**

|  | **Chi-square** | **df** | **Scaling correction factor** | **CFI** | **TLI** | **SRMR** | **RMSEA** |
| --- | --- | --- | --- | --- | --- | --- | --- |
| **Unconstrained models** |  |  |  |  |  |  |  |
| ***a) Education 🡪 AP*** | 64.246 | 21 | 1.0462 | 0.995 | 0.984 | 0.024 | 0.043 |
| ***b) AP 🡪 Education*** | 39.765 | 21 | 1.1312 | 0.998 | 0.994 | 0.015 | 0.028 |
| **Constrained models** |  |  |  |  |  |  |  |
| ***a) Education 🡪 AP*** | 67.735 | 28 | 1.0363 | 0.996 | 0.989 | 0.024 | 0.036 |
| ***b) AP 🡪 Education*** | 49.374 | 28 | 1.1072 | 0.998 | 0.995 | 0.017 | 0.026 |
|  |  |  |  |  |  |  |  |
| **Model comparison** | **Difference Test Scaling Correction (CD)** | | **Sattora-Bentler Scaled Chi-Square Difference (TRd)** | | **Difference is Degrees of Freedom (Δdf)** | | **P-value for TRd, Δdf** |
| ***a) Education 🡪 AP*** | 1.0066 |  | 2.9601 |  | 7 |  | 0.8887 |
| ***b) AP 🡪 Education*** | 1.0352 |  | 9.3554 |  | 7 |  | 0.2281 |

*Adjustment for time-invariant characteristics was performed by inclusion of a latent variable.*

*Edu = educational level; AP = attention problems; M = male; F = Female.*

***Boldface*** *denotes statistical significance at p < 0.05.*

*Parameters with different superscripts differ significantly from each other at p < 0.05, as determined by Wald Tests of Parameter Constraints.*

Potential gender differences in cross-lagged associations were investigated both by assessing gender differences in individual paths, using the ‘Model Test’ command in Mplus, as well as by comparing the fit of a model in which cross-lagged paths were constrained to be equal across genders to one allowing for gender differences in cross-lagged paths [2]. To test differences in fit between the constrained and unconstrained model, we applied the chi-square difference test using the Satorra-Bentler scaled chi-square [3].

**Fig. S10** Bidirectional associations between educational level and externalizing behaviour (EB) in males and females, in the TRAILS Study (the Netherlands, 2000–2017, N = 2,229); linear regression coefficients (stdyx-standardized ß-coefficient, robust standard error, p-value) from cross-lagged panel models with fixed effects

*
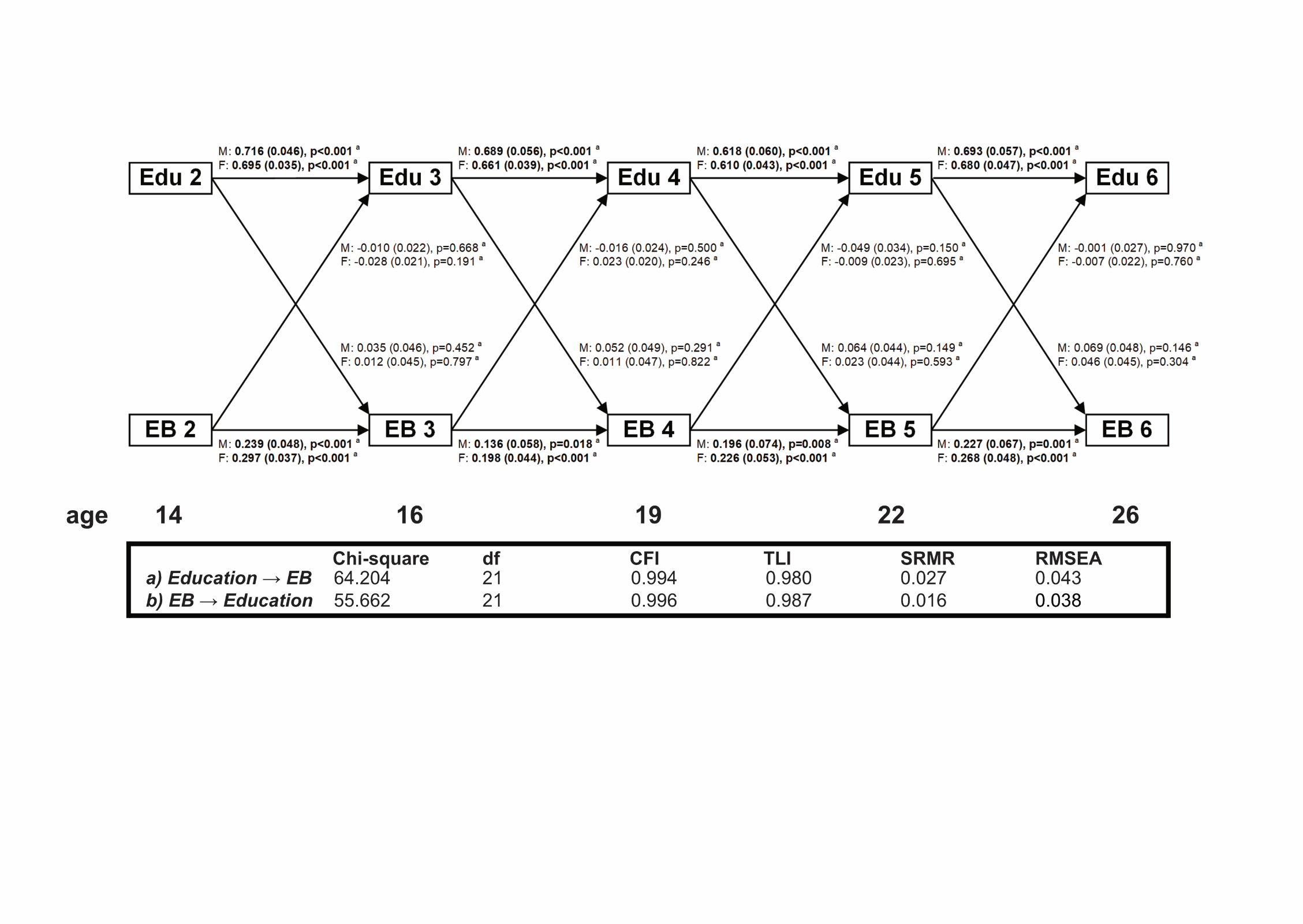
*

|  | **Chi-square** | **df** | **Scaling correction factor** | **CFI** | **TLI** | **SRMR** | **RMSEA** |
| --- | --- | --- | --- | --- | --- | --- | --- |
| **Unconstrained models** |  |  |  |  |  |  |  |
| ***a) Education 🡪 EB*** | 64.204 | 21 | 1.2193 | 0.994 | 0.980 | 0.027 | 0.043 |
| ***b) EB 🡪 Education*** | 55.662 | 21 | 1.0942 | 0.996 | 0.987 | 0.016 | 0.038 |
| **Constrained models** |  |  |  |  |  |  |  |
| ***a) Education 🡪 EB*** | 69.200 | 28 | 1.1702 | 0.994 | 0.986 | 0.028 | 0.036 |
| ***b) EB 🡪 Education*** | 62.113 | 28 | 1.0883 | 0.996 | 0.991 | 0.018 | 0.033 |
|  |  |  |  |  |  |  |  |
| **Model comparison** | **Difference Test Scaling Correction (CD)** | | **Sattora-Bentler Scaled Chi-Square Difference (TRd)** | | **Difference is Degrees of Freedom (Δdf)** | | **P-value for TRd, Δdf** |
| ***a) Education 🡪 EB*** | 1.0229 |  | 2.6336 |  | 7 |  | 0.9167 |
| ***b) EB 🡪 Education*** | 1.0706 |  | 6.2509 |  | 7 |  | 0.5108 |

*Adjustment for time-invariant characteristics was performed by inclusion of a latent variable.*

*Edu = educational level; EB = externalizing behaviour; M = male; F = Female.*

***Boldface*** *denotes statistical significance at p < 0.05.*

*Parameters with different superscripts differ significantly from each other at p < 0.05, as determined by Wald Tests of Parameter Constraints.*

Potential gender differences in cross-lagged associations were investigated both by assessing gender differences in individual paths, using the ‘Model Test’ command in Mplus, as well as by comparing the fit of a model in which cross-lagged paths were constrained to be equal across genders to one allowing for gender differences in cross-lagged paths [2]. To test differences in fit between the constrained and unconstrained model, we applied the chi-square difference test using the Satorra-Bentler scaled chi-square [3].

**Table S1** Attrition analysis – characteristics of young adults remaining in the TRAILS Study (the Netherlands, 2000–2017, N = 2,229) at wave 6, compared to participants who had dropped out of the cohort between wave 2 and wave 5

|  | **Participants remaining in TRAILS by wave 6** | | **Drop-outs** | | **P-value** |
| --- | --- | --- | --- | --- | --- |
| **N participants (%)** | 1,616 | (72.50) | 613 | (27.50) |  |
| **Baseline characteristics** |  |  |  |  |  |
| Male gender, N (%) | 735 | (45.48) | 363 | (59.22) | <0.001 |
| Non-Dutch ethnicity, N (%) | 155 | (9.59) | 146 | (23.82) | <0.001 |
| Age, mean (SD) | 11.09 | (0.56) | 11.16 | (0.54) | 0.006 |
| Parental socioeconomic status (SES), mean (SD) | 0.10 | (0.76) | -0.44 | (0.77) | <0.001 |
| Wechsler Intelligence Deviation Quotient (IQ), mean (SD) | 99.64 | (14.54) | 90.67 | (14.24) | <0.001 |
| **Educational level, mean (SD)** |  |  |  |  |  |
| *Wave 2* | 2.53 | (1.14) | 1.72 | (0.97) | <0.001 |
| *Wave 3* | 2.64 | (1.10) | 1.95 | (1.06) | <0.001 |
| *Wave 4* | 2.73 | (0.95) | 2.39 | (1.02) | <0.001 |
| *Wave 5* | 2.83 | (0.90) | 2.29 | (1.01) | <0.001 |
| **Attention problems, mean (SD)** |  |  |  |  |  |
| *Wave 2* | 0.56 | (0.34) | 0.56 | (0.33) | 0.864 |
| *Wave 3* | 0.59 | (0.35) | 0.62 | (0.33) | 0.227 |
| *Wave 4* | 0.45 | (0.32) | 0.49 | (0.34) | 0.088 |
| *Wave 5* | 0.43 | (0.32) | 0.45 | (0.32) | 0.639 |
| **Externalizing behaviour, mean (SD)** |  |  |  |  |  |
| *Wave 2* | 0.30 | (0.20) | 0.32 | (0.23) | 0.106 |
| *Wave 3* | 0.31 | (0.21) | 0.37 | (0.22) | <0.001 |
| *Wave 4* | 0.22 | (0.21) | 0.26 | (0.23) | 0.010 |
| *Wave 5* | 0.19 | (0.18) | 0.20 | (0.17) | 0.735 |

*SD = standard deviation.*

*P-values were computed using chi-squared tests for categorical variables and two-sample t-tests for continuous variables.*

**Table S2** Characteristics of participants with classifiable educational level compared to those with missing/unclassifiable educational level from wave 2 to 6 in the TRAILS Study (the Netherlands, 2000–2017, N = 2,229)

|  | **N participants per wave** |  | **Male gender** |  | **Non-Dutch ethnicity** |  | **Age at baseline** |  | **Parental**  **socioeconomic**  **status (SES)** | | **WechslerIntelli-gence Deviation Quotient (IQ)** | | **Concurrent attention problems** |  | **Concurrent exter-nalizing behaviour** |  |
| --- | --- | --- | --- | --- | --- | --- | --- | --- | --- | --- | --- | --- | --- | --- | --- | --- |
|  | **N** | **(%)** | **N** | **(%)** | **N** | **(%)** | **Mean** | **(SD)** | **Mean** | **(SD)** | **Mean** | **(SD)** | **Mean** | **(SD)** | **Mean** | **(SD)** |
| **Wave 2** | 2,148 | (100) |  |  |  |  |  |  |  |  |  |  |  |  |  |  |
| *Education complete* | 1,927 | (89.71) | 924 | (47.95) | 237 | (12.30) | 11.11 | (0.56) | -0.03 | (0.80) | 97.58 | (15.02) | 0.57 | (0.34) | 0.31 | (0.20) |
| *Education unclassifiable/missing* | 221 | (10.29) | 130 | (58.82) | 35 | (15.84) | 11.07 | (0.53) | -0.07 | (0.75) | 96.01 | (13.44) | 0.51 | (0.34) | 0.30 | (0.23) |
| *P-value* |  |  |  | 0.002 |  | 0.134 |  | 0.344 |  | 0.531 |  | 0.138 |  | 0.035 |  | 0.874 |
| **Wave 3** | 1,818 | (100) |  |  |  |  |  |  |  |  |  |  |  |  |  |  |
| *Education complete* | 1,529 | (84.10) | 704 | (46.04) | 175 | (11.45) | 11.09 | (0.56) | 0.08 | (0.78) | 99.75 | (14.85) | 0.60 | (0.34) | 0.32 | (0.21) |
| *Education unclassifiable/missing* | 289 | (15.90) | 163 | (56.40) | 36 | (12.46) | 11.19 | (0.57) | -0.32 | (0.78) | 91.25 | (13.22) | 0.57 | (0.36) | 0.33 | (0.22) |
| *P-value* |  |  |  | 0.001 |  | 0.623 |  | 0.004 |  | <0.001 |  | <0.001 |  | 0.323 |  | 0.489 |
| **Wave 4** | 1,880 | (100) |  |  |  |  |  |  |  |  |  |  |  |  |  |  |
| *Education complete* | 1,507 | (80.16) | 671 | (44.53) | 160 | (10.62) | 11.09 | (0.57) | 0.13 | (0.76) | 100.34 | (14.52) | 0.46 | (0.32) | 0.23 | (0.21) |
| *Education unclassifiable/missing* | 373 | (19.84) | 227 | (60.86) | 52 | (13.94) | 11.10 | (0.52) | -0.33 | (0.74) | 91.58 | (13.13) | 0.41 | (0.34) | 0.23 | (0.23) |
| *P-value* |  |  |  | <0.001 |  | 0.069 |  | 0.693 |  | <0.001 |  | <0.001 |  | 0.038 |  | 0.786 |
| **Wave 5** | 1,781 | (100) |  |  |  |  |  |  |  |  |  |  |  |  |  |  |
| *Education complete* | 1,429 | (80.24) | 624 | (43.67) | 147 | (10.29) | 11.10 | (0.56) | 0.12 | (0.76) | 100.13 | (14.68) | 0.43 | (0.32) | 0.19 | (0.18) |
| *Education unclassifiable/missing* | 352 | (19.76) | 219 | (62.22) | 52 | (14.77) | 11.11 | (0.55) | -0.23 | (0.76) | 93.78 | (13.88) | 0.46 | (0.35) | 0.25 | (0.20) |
| *P-value* |  |  |  | <0.001 |  | 0.017 |  | 0.608 |  | <0.001 |  | <0.001 |  | 0.310 |  | <0.001 |
| **Wave 6** | 1,616 | (100) |  |  |  |  |  |  |  |  |  |  |  |  |  |  |
| *Education complete* | 1,192 | (73.76) | 474 | (39.77) | 106 | (8.89) | 11.09 | (0.56) | 0.17 | (0.75) | 100.82 | (14.47) | 0.44 | (0.33) | 0.19 | (0.18) |
| *Education unclassifiable/missing* | 424 | (26.24) | 261 | (61.56) | 49 | (11.56) | 11.10 | (0.56) | -0.09 | (0.76) | 96.31 | (14.22) | 0.51 | (0.39) | 0.27 | (0.24) |
| *P-value* |  |  |  | <0.001 |  | 0.110 |  | 0.630 |  | <0.001 |  | <0.001 |  | 0.010 |  | <0.001 |

*SD = standard deviation.*

*P-values were computed using chi-squared tests for categorical variables and 2-sample t-tests for continuous variables.*

**Table S3** Amended scales with consistent items across YSR/ASR

| **YSR** | **ASR** |
| --- | --- |
| ***Attention problems*** |  |
| I fail to finish things that I start | I fail to finish things I should do |
| I have trouble sitting still | I have trouble sitting still |
| I have trouble concentrating or paying attention | I have trouble concentrating or paying attention for long |
| I feel confused or in a fog | I feel confused or in a fog |
| I daydream a lot | I daydream a lot |
| I act without stopping to think | I am impulsive or act without thinking |
| I’m too dependent on adults | I am too dependent on others |
| ***Externalizing behaviour*** |  |
| I argue a lot | I argue a lot |
| I am mean to others | I am mean to others |
| I try to get a lot of attention | I try to get a lot of attention |
| I destroy my own things | I damage or destroy my things |
| I destroy things belonging to others | I damage or destroy things belonging to others |
| I get in many fights | I get in many fights |
| I physically attack people | I physically attack people |
| I scream a lot | I scream or yell a lot |
| I am stubborn | I am stubborn, sullen, or irritable |
| My moods or feelings change suddenly | My moods or feelings change suddenly |
| I tease others a lot | I tease others a lot |
| I have a hot temper | I have a hot temper |
| I threaten to hurt people | I threaten to hurt people |
| I am louder than other kids | I am louder than others |
| I don’t feel guilty after doing something I shouldn’t | I don’t feel guilty after doing something I shouldn’t |
| I break rules at home, school, or elsewhere | I break rules at work or elsewhere |
| I hang around with kids who get in trouble | I hang around people who get in trouble |
| I lie or cheat | I lie or cheat |
| I would rather be with older kids than kids my own age | I would rather be with older people than with people of my own age |
| Mean score of “I steal at home” and “I steal from places other than home” | I steal |
| I cut classes or skip school | I stay away from my job even when I’m not sick or not on vacation |
| I brag | I brag |
| I show off or clown | I show off or clown |
| I talk too much | I talk too much |

**REFERENCES**

1. Allison PD, Williams R, Moral-Benito E (2017) Maximum likelihood for cross-lagged panel models with fixed effects. Socius 3:2378023117710578. <https://doi.org/10.1177/2378023117710578>

2. van Lier PAC, Vitaro F, Barker ED, Brendgen M, Tremblay RE, Boivin M (2012) Peer victimization, poor academic achievement, and the link between childhood externalizing and internalizing problems. Child Dev 83 (5):1775-1788. <https://doi.org/10.1111/j.1467-8624.2012.01802.x>

3. Muthén & Muthén (2021) Chi-square Difference testing using the Satorra-Bentler scaled chi-square. <https://www.statmodel.com/chidiff.shtml> Accessed 10th October 2021
